# Beyond body mass index: Body composition profiling for perioperative risk stratification in intrahepatic cholangiocarcinoma patients

**DOI:** 10.1002/cnr2.2070

**Published:** 2024-09-26

**Authors:** Johannes Kolck, Clarissa Hosse, Nick Lasse Beetz, Timo Alexander Auer, Adrian Alexander Marth, Laura Segger, Felix Krenzien, Georg Lurje, Uwe Pelzer, Dominik Geisel, Wenzel Schöning, Uli Fehrenbach

**Affiliations:** ^1^ Department of Radiology CKV, Charité – Universitätsmedizin Berlin Berlin Germany; ^2^ Berlin Institute of Health at Charité – Universitätsmedizin Berlin Berlin Germany; ^3^ Department of Surgery CCM/CVK Charité – Universitätsmedizin Berlin Berlin Germany; ^4^ Department of Hematology, Oncology and Cancer Immunology Charité – Universitätsmedizin Berlin Berlin Germany

**Keywords:** artificial intelligence, body composition analysis, computed tomography, intrahepatic cholangiocarcinoma, liver surgery, perisurgical complications

## Abstract

**Background and Aims:**

Intrahepatic cholangiocarcinoma (iCC) is an aggressive tumor, usually detected at an advanced stage. Our aim was to investigate the potential of body composition analysis (BCA) derived from presurgical staging computed tomography (CT) in predicting perisurgical complications.

**Methods:**

In this retrospective cohort study, we enrolled 86 patients who underwent CT imaging prior to liver surgery. Cox and logistic regression were performed to assess risk factors for prolonged hospital and intensive care unit (ICU) stays, as well as the occurrence of various complications. BCA parameters served as covariates besides conventional risk factors.

**Results:**

Postoperative complications after resection of iCC significantly prolonged the overall length of hospitalization (*p* < .001). Presence of sarcopenia was associated with longer ICU stays. Complications were common, with 62.5% classified as Clavien–Dindo grade IIIa or lower and 37.5% as more severe. Subcutaneous adipose tissue (SAT) and visceral adipose tissue (VAT) were identified as risk factors for complications, including bile leakage (in 24 cases, *p* = .025), pleural effusions (in 26 cases, *p* = .025), and intra‐abdominal abscess formation (in 24 cases, *p* = .043). SAT was associated with severe complications requiring interventional therapy, whereas VAT was correlated with abscess formation. Despite normal prevalence of obesity (22%), body mass index (BMI) did not have an impact on the development of perioperative complications.

**Conclusion:**

BCA is a useful tool for preoperative risk stratification in patients with iCC and is superior to BMI assessment. Increased SAT and VAT were associated with the risk of perisurgical complications, prolonging hospitalization. Therefore, BCA derived from routine staging CT should be considered in the preoperative assessment of patients with iCC.

**Abbreviations**
AI: artificial intelligence
ALLPS: associating liver partition with portal vein ligation for staged hepatectomy
BCA: body composition analysis
BMI: body mass index
CT: computed tomography
ICU: intensive care unit
PMI: psoas muscle index (psoas muscle area normalized to patient's height)
SMA: skeletal muscle area
SMI: skeletal muscle index (overall skeletal muscle normalized to patient's height)
SAT: subcutaneous adipose tissue
VAT: visceral adipose tissue

## 1 INTRODUCTION

Intrahepatic cholangiocarcinoma (iCC) is a type of cancer most commonly arising from the second‐order bile ducts in the liver.^1^, ^2^ It is a rare but an aggressive entity that accounts for approximately 10%–12% of all malignant liver tumors and most commonly occurs between the ages of 50 and 70 years.^3^ The incidence of iCC varies with certain geographic risk factors, particularly in Southeast Asia, where parasitic infections with *Opisthorchis viverrini* and *Clonorchis sinensis* are more frequent. In the Western world, primary sclerosing cholangitis is the most common risk factor.^4^, ^5^ Because of their often silent clinical presentation, iCCs are usually detected at advanced stages. Only about 35% of cases are eligible for primary surgical resection, which is considered a complex procedure, with reported morbidity rates ranging from 4.1% to up to 47.7%, but represents the mainstay of curative‐intended treatment for patients with iCC.^6^, ^7^ The risks of liver surgery include perisurgical bleeding and bile leakage, which can lead to subsequent complications, such as infection, abscess formation, and sepsis, and might prolong patient's hospitalization and recovery time.^8^, ^9^

In recent years, computed tomography (CT)‐derived body composition analysis (BCA) has become increasingly popular, as parameters like skeletal muscle quantification and its link to sarcopenia, have proven valuable in informing about frailty.^10^, ^11^ While CT imaging is frequently used for the staging of patients with iCC prior to surgery, the information CT provides on patients' body composition and physical status is not yet routinely used in clinical practice.^12^ Although surgical treatments are alike, the patient demographics of iCC diverge from those of hepatocellular carcinoma (HCC) in terms of gender distribution and the prevalence of preexisting liver and general conditions such as cirrhosis and cachexia, and should therefore be considered separately.^13^

Therefore, we conducted a study to investigate the potential of BCA metrics for predicting the occurrence of complications in patients undergoing liver surgery for iCC in order to identify possible patient‐specific risk factors.

## 2 METHODS

### 2.1 Study design

In this single‐center cohort study, we analyzed body composition from presurgical CT of patients with histologically proven iCC who underwent curative‐intended liver surgery. The study was conducted according to the guidelines of the Declaration of Helsinki and was approved by the Institutional Review Board (Internal registration number: EA4/152/20). Informed consent was waived by the ethics committee, in accordance with Section 25 of the Berlin State Hospital Act.

### 2.2 Patient population, data collection, and endpoints

All patients were admitted for curative‐intended liver surgery to the Department of Visceral Surgery of Charité—Universitätsmedizin Berlin between March 2005 and August 2018. Inclusion criteria were histologically proven iCC, availability of presurgical contrast‐enhanced CT datasets, clinical data including the presence or absence of the following complications: bile leakage, insufficiency or stenosis of anastomosis, postoperative bleeding, portal venous thrombosis, intra‐abdominal abscess, pleural effusion, pneumonia, and postoperative kidney and liver failure. All data were obtained from patient records and the clinical database. Body mass indices (BMI) were calculated. Clinical endpoints were defined as length of hospital and ICU stays, as well as occurrence and need for interventional treatment of complications.

### 2.3 Body composition analysis

**FIGURE 1:**
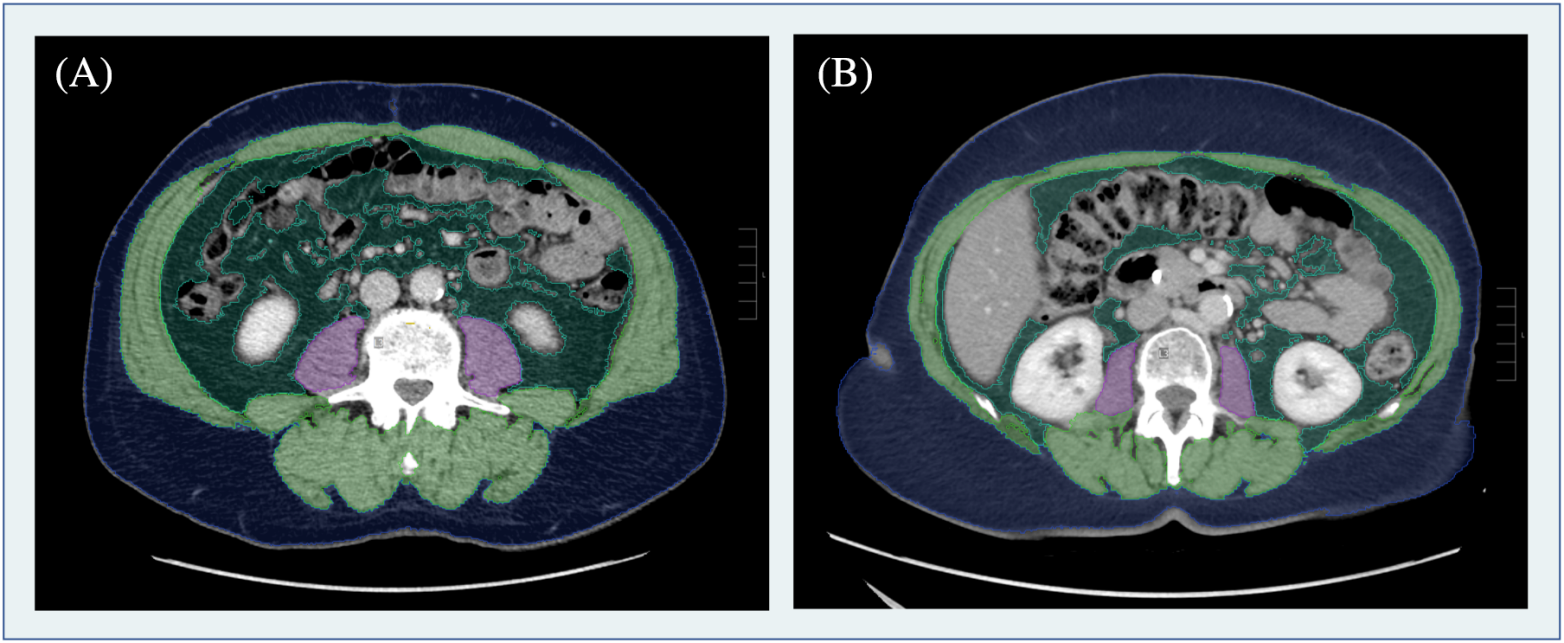
Two examples of automated PACS‐integrated body composition analysis. Each segmented tissue is coded with a different color: psoas muscle = purple, skeletal muscle (without psoas muscle) = green, visceral fat = dark green, and subcutaneous fat = blue. Tissue density and area are automatically calculated using Visage version 7.1. Even though the two patients have the same body mass index of 30, their body composition analysis reveals obvious differences in tissue distribution, with patient (A) showing a relatively greater muscle area, whereas patient (B) has a higher proportion of subcutaneous adipose tissue.

CT imaging datasets were either acquired at or transmitted to the Department of Radiology. BCA was performed using an artificial intelligence (AI)‐based image segmentation tool, which has previously been applied in several studies.^11^, ^14^ The tool is based on a convolutional neural network. It is integrated into a commonly used Picture Archiving and Communication System software (Visage version 7.1., Visage Imaging GmbH, Berlin, Germany). The tool recognizes the L3 lumbar vertebra and automatically segments the different tissue components on this level. Visceral adipose tissue, subcutaneous adipose tissue, psoas muscle, and skeletal muscle areas (SMAs) are automatically coded with different colors. For skeletal muscle, psoas muscle, visceral adipose tissue (VAT), and subcutaneous adipose tissue (SAT), the area in square centimeters (cm^2^) and density in Hounsfield units are automatically calculated. In our study, an experienced radiologist checked the results of automatic segmentation and identified no case that required manual correction. The psoas muscle index (PMI) and skeletal muscle index (SMI) were calculated using the following formulas: PMI = psoas muscle area (cm^2^)/body surface area (m^2^) and SMI = SMA without psoas muscle area (cm^2^)/body surface area (m^2^). An example of AI‐based automated analysis of body composition in two iCC patients is presented in Figure 1.

### 2.4 Clavien–Dindo classification and need for intervention

The Clavien–Dindo classification is a system for grading the severity of complications or adverse events occurring after surgical procedures. It was developed in 2004 and aims to standardize the reporting of complications in surgical research and clinical practice. The Clavien–Dindo system has become widely used in surgery and is considered a useful tool for evaluating the safety and effectiveness of surgical procedures. The classification system consists of five categories, numbered from 1 to 5, with each grade representing a progressively more severe degree of complication (Table 1).^15^

**TABLE 1:** Grades of Clavien–Dindo classification for postsurgical complications.

Grade	Definition
I	Any deviation from the normal postoperative course without the need for pharmacological treatment or surgical, endoscopic, or radiological interventions. Allowed therapeutic regimens include drugs such as antiemetics, antipyretics, analgesics, diuretics, electrolytes, and physiotherapy. This grade also includes wound infections opened at the bedside.
II	Requirement for pharmacological treatment with drugs other than those allowed for grade I complications, including blood transfusions and total parenteral nutrition.
III	Requiring surgical, endoscopic, or radiological intervention.
IIIa	Intervention not under general anesthesia.
IIIb	Intervention under general anesthesia.
IV	Life‐threatening complication requiring IC/ICU management.
IVa	Single‐organ dysfunction.
IVb	Multiorgan dysfunction.
V	Death of the patient.

Abbreviation: ICU, intensive care unit.

### 2.5 Statistical analysis

Descriptive statistics for all numeric and categorical variables were calculated as mean or median. Cutoffs for sarcopenia, defined as SMI ≤38.5 cm^2^/m^2^ in females and SMI ≤52.4 cm^2^/m^2^ in males, and obesity, defined as BMI ≥30 kg/m^2^, were used.^16^ Patients who were both sarcopenic and obese were classified as having sarcopenic obesity.

For risk stratification, lengths of hospital and ICU stays served as a time variable for Cox logistic regression analysis. Sex, age, BMI, occurrence of complications, and the AI‐derived body composition parameters—SMI, VAT, and SAT—as well as presence of sarcopenia and sarcopenic obesity were used as variates. Kaplan–Meier curves for length of hospital stay were calculated for patients with and without complications. For binary logistic regression analysis, the dependent variable was defined as occurrence or absence of complications greater or equal to Clavien–Dindo grade IIIb, occurrence or absence of specific complications including bile leakage, insufficiency or stenosis of anastomosis, postoperative bleeding, portal venous thrombosis, intra‐abdominal abscess, pleural effusion, pneumonia, and postoperative kidney and liver failure. Age at surgery, gender, and BMI, as well as BCA‐derived metrics, SAT, VAT, PMI, and SMI, served as variates. Data analyses were performed using IBM SPSS Statistics version 27 (IBM, Armonk, New York, USA). All *p*‐values <.05 were considered statistically significant.

## 3 RESULTS

### 3.1 Baseline data

A total of 86 patients with iCC admitted for curative‐intended surgical resection were retrospectively included in this study. Mean age at the time of surgery was 64 years (range of 32–82 years). There were 41 females (48%) and 45 males (52%). The mean weight was 74 ± 15 kg, the mean height was 170 ± 8 cm, and mean BMI was 25 ± 4.8 kg/m^2^.

### 3.2 Preconditions

Fifty‐nine patients (69%) had sarcopenia based on AI‐derived cutoffs. Twenty‐four patients (28%) had a BMI > 25, but <30, and 19 patients' BMI exceeded 30 (22%). The total proportion of patients with overweight was thus 50%. A minority of 11 patients (13%) showed the combination of both and was classified as sarcopenic obese. The vast majority of patients (62 out of 86) had no significant liver conditions prior to the diagnosis of iCC. Among the minority, 19 patients exhibited liver fibrosis, while two patients presented with hepatitis. Neoadjuvant chemotherapy prior to surgery was administered to only five out of 86 patients.

### 3.3 Liver resection and neoadjuvant treatment

The goal of curative‐intended surgery is to achieve complete margin‐negative resection (R0) with a sufficient future liver remnant. Most patients in our study had large iCC tumors with a median diameter of 7.2 cm (interquartile range 5.3–9.0 cm). Presurgical biopsies were obtained in 41 cases. Thirteen patients underwent portal venous embolization, and three had associating liver partition with portal vein ligation for staged hepatectomy (ALLPS) to induce hypertrophy of the healthy liver tissue remaining after surgery (future liver remnant). The most commonly performed surgery was hemihepatectomy, with 24 cases on the left and 14 cases on the right liver lobes, along with extended right hemihepatectomy in 15 cases and left hemihepatectomy in 3 cases. Trisectorectomy was carried out in 21 cases, with 14 instances involving the right liver and 7 the left liver. Fourteen patients underwent other types of surgery, including left lateral resection of segments 2 and 3. The majority of patients underwent (extended) lymphadenectomy, with at least six hilar lymph nodes removed. Nearly half (49 out of 86) of the patients received either Neuhaus or T‐drainages during surgery. Only three patients required transfusion of two units of red blood cells each due to intraoperative bleeding. Laparoscopic surgery was performed in only four cases. Specialized liver surgeons with consistent levels of clinical experience conducted all operations. Neither the type of surgery nor the placement of drainages was statistically significantly associated with the occurrence of complications. Relevant clinical patient characteristics are compiled in Table 2.

**TABLE 2:** Clinical characteristics of patients undergoing curative‐intended liver surgery for iCC.

	Total (*n* = 86)
Age, years	64 ± 11
Sex, *n* (%)	
Female	41 (37%)
Male	45 (63%)
BMI	25.6 ± 4.8
Precondition, *n* (%)	
Sarcopenia*	59 (69%)
Obesity (BMI > 30)	19 (22%)
Sarcopenic obesity**	11 (13%)
Type of surgery	
Right HH	14 (16%)
Left HH	24 (28%)
Extended right HH	15 (17%)
Extended left HH	3 (3%)
Right trisectorectomy	14 (16%)
Left trisectorectomy	7 (8%)
Other	14 (16%)

Abbreviations: BMI, body mass index; HH, hemihepatectomy; iCC, intrahepatic cholangiocarcinoma; SMI, skeletal muscle index.

* Cutoffs for sarcopenia are defined as SMI ≤38.5 cm^2^/m^2^ in females and SMI ≤52.4 cm^2^/m^2^ in males.

** Presence of both sarcopenia and obesity.

### 3.4 AI‐derived body composition parameters

All AI‐based body composition parameters were derived at the third lumbar vertebra level. Mean SMA was 114.15 ± 33 mm^2^, mean VAT was 134.14 ± 103 mm^2^, mean SAT was 162 ± 85.52 mm^2^, and mean PMA was 13.48 ± 6.27 mm^2^. The mean SMI (without psoas muscle) was 39.04 ± 9.95 cm^2^/m^2^, the mean PMI was 4.55 ± 1.88 cm^2^/m^2^, and the SMI including PMI was 43.60 ± 11–26 cm^2^/m^2^.

### 3.5 Clavien–Dindo classification and occurrence of complications

Overall, 22 patients had surgery without any complications. Sixty‐four patients developed complications, of which the majority (40; 62.5%) were classified as Clavien–Dindo grade IIIa or lower. More severe complications occurred in 24 patients (37.5%). The most common Clavien–Dindo grades were IIIa, II, and IIIb with 20, 13, and 10 incidences, respectively. Bile leakage occurred in 24 patients and was treated by endoscopic stenting or percutaneous drain insertion in 19 cases and relaparotomy in 5 cases. Biliary anastomotic insufficiency occurred in 13 cases, whereas stenosis was present in only two patients. Postoperative bleeding occurred in three cases, and portal vein thrombosis in one patient. Intra‐abdominal abscess formation was more frequent (24 of 86 patients), as was pleural effusion (26 of 86 patients), distantly followed by pneumonia (14 of 86 cases). Postoperative renal and hepatic failure was observed in 11 and 8 cases, respectively. An overview of the specific complications is given in Table 3.

**TABLE 3:** Overview of complications after liver surgery on intrahepatic cholangiocarcinoma patients.

Complications	Incidences
Bile leakage	24 (27.9%)
Endoscopic stenting or radiological drainage	19 (22.0%)
Relaparotomy	5 (0.6%)
Biliary anastomotic insufficiency	13 (15.1%)
Biliary anastomotic stenosis	2 (2.3%)
Postoperative bleeding	3 (3.4%)
Portal vein thrombosis	1 (1.1%)
Intra‐abdominal abscesses	24 (27.9%)
Postoperative renal failure	11 (12.8%)
Postoperative liver failure	8 (9.3%)
Pleural effusion	26 (30.2%)
Pneumonia	14 (16.3%)

*Note*: Some patients had more than one complication.

### 3.6 Lengths of hospital and ICU stays

Eighty‐two (95%) patients were referred to the ICU after liver surgery. The mean length of ICU stay was 7 days, ranging between 1 and 94 days. The mean length of overall hospital stay was 34 days, ranging from 6 to 98 days. Nine patients (10%) died during hospitalization, these were excluded from risk stratification for prolonged hospital stays.

### 3.7 Risk stratification for prolonged hospital and ICU stays

**FIGURE 2:**
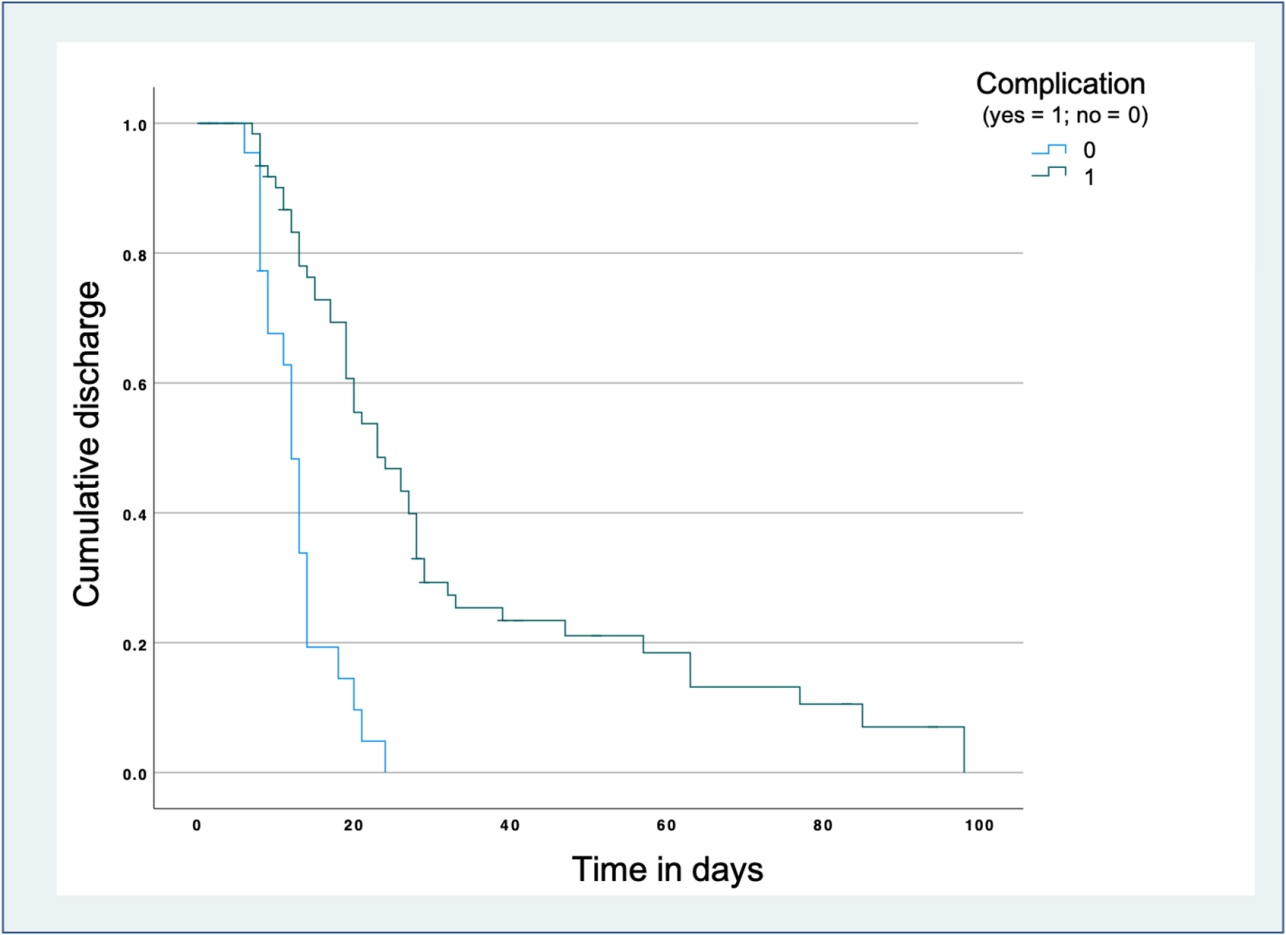
Kaplan–Meier curves of hospital length of stay of patients with and without postoperative complications after curative‐intended liver surgery. Hospitalization is significantly prolonged in patients with complications (*p* < .001).

Calculation of Kaplan–Meier curves revealed significantly longer hospital stays for patients with postsurgical complications (*p* < .001), as depicted in Figure 2. Thus, the occurrence of complications was an obvious, strong predictor of both prolonged ICU and hospital stays (*p* = .017; *p* < .001). Moreover, presence of sarcopenia proved to be an independent predictor of prolonged ICU stays (*p* = .029) but not of total length of hospital stay. Age at surgery, gender, BMI, presence of sarcopenic obesity, and BCA‐derived metrics (SAT, VAT, and SMI) showed no predictive influence. The results are compiled in Table 4.

**TABLE 4:** Cox regression analysis of patients with iCC.

	Length of ICU stay		Length of hospital stay	
Variate	*p*‐value	Odds ratio (CI)	*p*‐value	Odds ratio (CI)
Age	.969	0.999 (0.974–1.025)	.952	1.001 (0.976–1.026)
Gender	.450	1.300 (0.658–2.569)	.829	0.930 (0.482–1.797)
BMI	.951	1.004 (0.889–1.133)	.426	0.950 (0.836–1.078)
Sarcopenia	**.029**	0.369 (0.151–0.905)	.574	0.774 (0.317–1.888)
Sarcopenic obesity	.640	0.798 (0.310–2.055)	.448	0.689 (0.263–1.803)
SMI	.328	0.975 (0.926–1.026)	.669	1.012 (0.960–1.066)
VAT	.474	0.998 (0.994–1.003)	.805	1.000 (0.997–1.004)
SAT	.355	1.002 (0.997–1.007)	.282	1.003 (0.998–1.008)
Complications	**.017**	0.475 (0.257–0.877)	**<.001**	0.227 (0.116–0.443)
Number	82		77	

*Note*: Sex, age, BMI, sarcopenia, sarcopenic obesity, AI‐derived body composition parameters, and occurrence of postsurgical complications were used as variates. Significant *p*‐values (*p* < 0.05) are printed in bold.

Abbreviations: BMI, body mass index; CI, confidence interval; iCC, intrahepatic cholangiocarcinoma; SAT, subcutaneous adipose tissue; SMI, skeletal muscle index; VAT, visceral adipose tissue.

### 3.8 Risk factors for complications and the need for interventional treatment

AI‐derived BCA metrics, SAT and VAT, were identified as risk factors for a number of complications occurring after liver surgery. Elevated SAT, representing subcutaneous fat tissue, and VAT, representing visceral fat tissue, are risk factors for the occurrence of postsurgical complications in general (*p* = .010 and *p* = .015, respectively). Furthermore, elderly patients and those with elevated SAT have a higher risk of suffering complications that require interventional treatment (Clavien–Dindo grades III–V, *p* = .010). Elevated SAT was also identified as a risk factor for bile leakage requiring radiological or endoscopic intervention (*p* = .025) and for the development of pleural effusions (*p* = .025). VAT represents a risk factor for intra‐abdominal abscess formation (*p* = .043), and female sex was associated with a higher risk of postoperative liver failure (*p* = .040). No risk factors for the occurrence of anastomotic insufficiency or stenosis, postoperative bleeding, pneumonia, or kidney failure were identified. BMI and BCA‐derived metrics, PMI and SMI, were not found to have an effect on the occurrence of complications. The results of binary logistic regression analysis are compiled in Table 5.

**TABLE 5:** Logistic regression analysis of iCC patients for assessment of risk factors for complications after curative‐intended surgery.

	Complications in general		Clavien–Dindo III–V		Bile leakage	
	*p*	Odds ratio (CI)	*p*	Odds ratio (CI)	*p*	Odds ratio (CI)
Gender	.094	3.462 (0.810–14.803)	.875	0.996 (0.949–1.046)	.898	0.996 (0.938–1.058)
Age	.769	1.009 (0.953–1.067)	**.045**	3.620 (1.031–12.708)	.814	1.230 (0.218–6.940)
BMI	.298	0.878 (0.687–1.122)	.288	0.893 (0.725–1.100)	.283	0.882 (0.701–1.110)
VAT	**.015**	0.989 (0.980–0.998)	.065	0.993 (0.985–1.000)	.664	1.002 (0.993–1.011)
SAT	**.010**	1.017 (1.004–1.031)	**.010**	1.014 (1.003–1.024)	**.025**	1.015 (1.002–1.028)
PMI	.354	0.804 (0.506–1.276)	.071	0.704 (0.481–1.030)	.061	1.705 (0.976–2.977)
SMI	.061	1.113 (0.995–1.244)	.052	1.093 (0.999–1.195)	.052	0.889 (0.789–1.001)

	Abscess		Pleural effusion		Liver failure	
	*p*	Odds ratio (CI)	*p*	Odds ratio (CI)	*p*	Odds ratio (CI)
Gender	.149	2.762 (0.694–10.992)	.095	3.331 (0.811–13.672)	**.040**	35.203 (1.167–1061.6)
Age	.613	1.013 (0.963–1.065)	.629	0.987 (0.938–1.039)	.673	1.020 (0.931–1.117)
BMI	.159	0.857 (0.691–1.062)	.121	0.841 (0.675–1.047)	.210	0.783 (0.534–1.148)
VAT	**.043**	0.991 (0.982–1.000)	.424	0.997 (0.989–1.005)	.217	0.989 (0.972–1.007)
SAT	.074	1.010 (0.999–1.020)	**.025**	1.012 (1.002–1.023)	.200	1.014 (0.992–1.037)
PMI	.996	0.999 (0.667–1.496)	.319	0.823 (0.562–1.207)	.084	0.547 (0.276–1.084)
SMI	.197	1.060 (0.970–1.158)	.119	1.072 (0.982–1.169)	.116	1.130 (0.970–1.315)

*Note*: Gender, age, BMI, and AI‐derived body composition metrics (VAT, SAT, PMI, and SMI) served as variates. Significant *p*‐values (*p* < 0.05) are printed in bold.

Abbreviations: BMI, body mass index; CI, confidence interval; iCC, intrahepatic cholangiocarcinoma; PMI, psoas muscle index; SAT, subcutaneous adipose tissue; SMI, skeletal muscle index; VAT, visceral adipose tissue.

## 4 DISCUSSION

Intrahepatic mass‐forming cholangiocarcinoma is a rare but aggressive liver malignancy that is mostly diagnosed in advanced stages. Until now, liver resection is the mainstay of curative‐intended therapy. However, (extended) hemihepatectomy is a complex surgical procedure with a high rate of perisurgical complications. In this study, we performed BCA on presurgical CT scans of patients scheduled for liver surgery to determine the potential of body composition metrics for preoperative identification of patient‐specific risk factors for complications.

In our study cohort, postoperative complications significantly prolonged the overall length of hospital stay and patients with sarcopenia were at higher risk of longer ICU stays. Moreover, the BCA‐derived metrics SAT and VAT were identified as risk factors for the occurrence of complications in general. Patients with higher amounts of subcutaneous fat were more likely to suffer severe complications requiring interventional therapy (Clavien–Dindo grades III–V), bile leakage, and pleural effusions, whereas VAT was associated with intra‐abdominal abscess formation. Higher age at surgery is a risk factor for severe complications (Clavien–Dindo III–V), whereas female gender was associated with the risk of liver failure. Despite the relatively normal prevalence of obesity (19 of 86 patients, 22%), BMI did not have a predictive impact on perioperative complications.

Body composition profiling, mainly focusing on sarcopenia, is gaining importance as a preoperative factor that is useful in predicting short‐ and long‐term outcomes for patients undergoing liver surgery for cancer. While the association between body composition and short‐term outcomes after liver resection for HCC has been investigated in several studies, data on the correlation with respect to cholangiocarcinoma are clearly limited.^17^ To date, the influence of body composition on short‐term outcomes after liver surgery for iCC and perihilar cholangiocarcinoma has been investigated in only four studies.^18^, ^19^, ^20^, ^21^ Three of these studies identified sarcopenia, or reduced muscle mass, as a risk factor for liver failure and serious morbidity. However, only one study found an association between low skeletal muscle mass and increased postoperative mortality, and the results of the study by Ardito et al. were not statistically significant.^18^, ^19^, ^21^ The fourth study examined the impact of skeletal muscle mass/quality and visceral adiposity on hepatectomy outcomes in iCC, with no significant differences in overall morbidity and mortality in iCC patients.^20^

A more robust association has been established between obesity and an increased risk of potentially life‐threatening morbidity after major liver surgery. Obese patients were reported to have longer pedicular clamping, more frequently developed severe complications (Clavien–Dindo grade III, or higher), and required longer ICU and hospital stays.^22^, ^23^ Congruently, Zhou et al. were able to identify the BMI as an independent risk factor for higher postoperative morbidity in patients undergoing surgical treatment for cholangiocarcinoma.^24^ However, the common definition of obesity is based on a patient's BMI, which neglects the distribution of muscle and adipose tissues that contribute to a patient's body weight and thus BMI. The example in Figure 1 nicely illustrates how tissue distribution can differ in patients with the same BMI. Consequently, a risk assessment based on BMI alone attributes the same risk to supposedly fit patients with a relatively high proportion of muscle mass as to patients with the same BMI but considerably more adipose tissue. A meta‐analysis of 31 968 individuals investigating the diagnostic performance of BMI showed that, compared with direct fat measures, BMI had a high specificity (0.90), but relatively low sensitivity of only 0.50 for the assessment of obesity.^25^ In contrast, BCA enables precise quantification of tissue compartments and the identification of sarcopenia, characterized by reduced muscle strength or muscle mass decline. Patients with sarcopenia are generally at risk for cancer cachexia, marked by weight and muscle mass loss and/or low BMI, which correlates with heightened chemotherapy toxicity and a grim prognosis. The interaction between physiological sarcopenia and cancer cachexia contributes significantly to the intricacies of studying wasting disorders in geriatric oncology and needs further investigation.^26^ More specifically, sarcopenia has also been identified as a risk factor for long‐term outcomes in iCC and is associated with risks such as prolonged ICU stays.^27^, ^28^ Our results demonstrate that BCA‐derived metrics not only enable identification of patient‐specific risk factors for surgical complications independently of BMI but even identify at‐risk patients who would be missed by risk stratification based on BMI alone. As preoperative imaging is usually performed weeks to months before resection, translation of BCA into routine clinical practice would inform both patients and surgeons of potential risks and even allow the initiation of preventive measures before scheduled surgery. A recent study of Halliday et al. highlights the great potential of prehabilitation. The authors showed that physical exercise in patients with esophageal cancer led to a sufficient reduction of patients' adipose tissue and thereby significantly reduced postsurgical complications.^29^

### 4.1 Limitations

To improve the value of BCA in routine clinical practice, further studies should be conducted to establish standardised, age‐specific thresholds for SAT and VAT. Moreover, generalizability and validity of our results are limited by the moderate size of our single‐center study population, which is attributable to the low prevalence of intrahepatic cholangiocarcinoma. The impact of BMI might have been underestimated due to the small size of our study population. In line with existing literature, sarcopenic patients exhibited prolonged stays in the intensive care unit; however, no significant difference in total length of stay compared with nonsarcopenic counterparts was observed. This lack of distinction may be attributed to potential subsequent stays in rehabilitation clinics, a factor not accounted for in our study.

### 4.2 Conclusion

BCA provides a more precise risk assessment for perioperative complications in patients with iCC compared with assessment based on BMI alone. Sarcopenia is linked to longer ICU stays, while quantifying SAT and VAT can identify patients at risk for major complications. By improving risk stratification and informing patients and surgeons of potential risks, BCA‐derived metrics have the potential to facilitate the initiation of preventive measures before scheduled surgery.

## AUTHOR CONTRIBUTIONS

UF and DG provided conception and design. FK, GL, UP, WS and UF provided clinical data andguidance on data analysis and interpretation. CH, JK, UF and DG conducted the statistical analysis. CH and JK wrote the manuscript. NLB, TAA, AAM and LS contributed with critical revision of the manuscript.

## CONFLICT OF INTEREST STATEMENT

The authors have stated explicitly that there are no conflicts of interest in connection with this article.

## ETHICS STATEMENT

The study was conducted according to the guidelines of the Declaration of Helsinki and was approved by the Institutional Review Board (Internal registration number: EA4/152/20).

## Data Availability

The data supporting the findings of this study are available upon reasonable request.
